# Comparative Analysis of Bilateral-Based Noise Reduction Algorithms in Low-Dose Pediatric Computed Tomographic Images

**DOI:** 10.3390/diagnostics16193186

**Published:** 2026-09-30

**Authors:** Minji Park, Hajin Kim, Sang Woong Park, Min-Hee Lee, Youngjin Lee

**Affiliations:** 1 Department of Health Science, General Graduate School of Gachon University, 191, Hambangmoe-ro, Yeonsu-gu, Incheon 21936, Republic of Korea; mu05430@gachon.ac.kr (M.P.); happida3@gachon.ac.kr (H.K.); 2 Department of Emergency Medical Services, Eulji University, 553, Sanseong-Daero, Sujeong-gu, Seongnam-si 13135, Republic of Korea; swpark@eulji.ac.kr; 3 Department of Pediatrics, Wayne State University School of Medicine, 4201 St Antoine 10 Boulevard, Detroit, MI 48201, USA; 4 Department of Radiological Science, Gachon University, 191, Hambangmoe-ro, Yeonsu-gu, Incheon 21936, Republic of Korea

**Keywords:** low-dose pediatric abdominal computed tomography, bilateral-based noise reduction algorithm, visual assessment, quantitative measurement, statistical comparison, noise reduction, structural preservation

## Abstract

**Background:** Noise reduction in low-dose pediatric computed tomography (CT) requires effective suppression of quantum noise without compromising subtle anatomical structures. This study compared three bilateral-based noise reduction algorithms, namely the conventional bilateral filter (BF), adaptive BF (ABF), and trainable BF (TBF), using eight low-dose pediatric abdominal CT cases. **Methods:** Denoising performance was evaluated in the axial and coronal planes through visual assessment and quantitative measurements of the coefficient of variation (CV) within the liver parenchyma and the contrast-to-noise ratio (CNR) between the liver and portal vein. Statistical comparisons were performed using the Friedman test, followed by the Wilcoxon signed-rank tests with Holm-adjustment. All three methods reduced the visible noise present in the original images. **Results:** ABF improved uniformity in homogeneous liver regions while preserving anatomical detail, whereas TBF produced the strongest apparent noise suppression but introduced greater blurring and occasional ambiguous structures. Quantitatively, the CV decreased progressively from BF to ABF and TBF. The TBF method achieved the lowest average CV and the highest average CNR. Significant differences were observed among the four conditions for both metrics in each imaging plane. However, CNR differences between the three denoising methods were less consistent, particularly for comparisons involving TBF. **Conclusions:** Overall, ABF provided a favorable balance between noise reduction and structural preservation, whereas TBF offered stronger image specific denoising with additional concerns regarding signal fidelity and reproducibility. Further refinement is required to ensure clinical reliability without compromising quantitative accuracy or anatomical details.

## 1. Introduction

Computed tomography (CT) is one of the most valuable diagnostic imaging modalities in modern medicine, providing high-resolution three-dimensional anatomical information with excellent bone-to-soft tissue contrast [1,2,3]. However, these diagnostic advantages are inherently accompanied by exposure to ionizing radiation. This concern is particularly pronounced in pediatric patients because developing tissues exhibit greater radiosensitivity and the longer life expectancy provides a wider temporal window for the manifestation of radiation-induced malignancies [4,5,6]. A multinational cohort study that followed approximately 950,000 individuals who underwent CT examinations before the age of 22 demonstrated a consistent dose-dependent increase in the risk of hematologic malignancies even at radiation doses typically associated with CT examinations [7,8]. This body of evidence has established the foundation for the principle of ‘As Low As Reasonably Achievable’ (ALARA) in pediatric CT [9]. Furthermore, modeling studies have suggested that reducing high-dose examinations to a moderate dose could prevent a substantial proportion of future CT-related cancers [10,11].

The most direct approach to achieving the ALARA principle is to reduce radiation dose by lowering the tube current or tube voltage [12]. However, decreasing the photon flux inevitably increases mixed Poisson–Gaussian noise, thereby degrading low-contrast detectability and obscuring small anatomical structures [13,14,15]. Although model-based iterative reconstruction (IR) algorithms have been developed to mitigate this noise directly during image formation, their clinical utility remains limited by vendor-specific implementation, substantial computational demands, and the need for access to raw projection data, which restricts their applicability to reconstructed DICOM images already in clinical use [16,17,18]. Consequently, effective post-acquisition denoising has become an essential component of low-dose CT imaging to reconcile aggressive dose reduction with the image quality required for reliable diagnosis [19,20]. An ideal denoising algorithm should suppress noise in homogeneous regions while preserving diagnostically important edges, fine textures, and low-contrast lesions [21].

Among edge-preserving denoising techniques, the bilateral filter (BF), originally proposed by Tomasi et al., has become one of the most widely adopted approaches [22]. The BF combines a spatial Gaussian kernel with an intensity-domain Gaussian kernel, restricting the averaging process to neighboring pixels that are both spatially proximal and similar in intensity [23]. This mechanism effectively smooths homogeneous regions while preserving sharp intensity transitions. BF remains an attractive baseline denoising algorithm owing to its conceptual simplicity and noniterative implementation. However, the conventional BF relies on globally fixed spatial and range parameters, preventing adaptation to spatially varying noise characteristics and heterogeneous image structures [24]. This limitation is associated with staircasing artifacts and gradient reversal and imposes an inherent trade-off between noise suppression and edge preservation [25].

Adaptive BF (ABF) was subsequently introduced to overcome the limitations associated with fixed parameters [26]. ABF spatially modulates the range kernel by adaptively adjusting its bandwidth and incorporating a locally varying offset, thereby simultaneously enhancing edge sharpness and suppressing image noise [27,28]. ABF provides improved detail and edge preservation compared the conventional BF [29]. Nevertheless, the adaptive approach remains governed by manually designed heuristics or pre-optimized parameters and therefore may not be optimal for the heterogeneous noise distributions commonly encountered in low-dose CT imaging [30]. More recently, deep learning-based denoising methods have achieved state-of-the-art performance in low-dose CT denoising [31,32,33]. However, these approaches typically contain millions of trainable parameters and operate as opaque black-box models, raising concerns regarding prediction instability and the potential generation of anatomically nonexistent structures [34]. These limitations are particularly problematic in diagnostic imaging, where data fidelity is of paramount importance [35].

The trainable BF (TBF) embeds the classical bilateral filtering operation into a fully differentiable framework and directly optimizes a small set of trainable parameters, namely three spatial parameters and one range parameter per layer, using gradient-based learning [36]. The design of TBF preserves the interpretability and well-defined behavior of conventional BF while achieving denoising performance comparable to that of computationally intensive deep neural network-based approaches [37]. Furthermore, extended TBF frameworks that exploit paired ground truth images during supervised learning have demonstrated improved prediction stability. Consequently, TBF occupies an intermediate position between manually designed classical filters and highly parameterized deep neural networks [38].

Conventional BF, ABF, and TBF represent a natural evolutionary progression of the same edge-preserving filtering paradigm, advancing from fixed parameterization to heuristic adaptation and ultimately to data-driven optimization. The relative performance of these three algorithms has not been systematically evaluated in the context of low-dose pediatric CT imaging despite their clear conceptual relationship. Pediatric CT presents unique challenges because of the smaller body size of patients, inherently lower tissue contrast, and stringent requirements for preserving fine anatomical structures [39,40]. Nevertheless, most previous evaluations have relied on adult phantoms or adult abdominal benchmark datasets.

Therefore, the present study was designed as an exploratory methodological comparison. We performed a fully paired within-case comparison of three bilateral filter-based denoising strategies on low-dose pediatric CT images by quantitatively evaluating both image quality and structural preservation. The objective of this study was to characterize the trade-off between noise suppression and structural preservation for each method. We also aimed to provide preliminary insights to inform the selection of a bilateral filter-based denoising strategy for low-dose pediatric CT imaging rather than to establish definitive clinical recommendations.

## 2. Materials and Methods

### 2.1. Acquisition of Low-Dose Pediatric Abdominal Computed Tomographic Images

The low-dose pediatric CT images used in this study were obtained from an open-access dataset available through The Cancer Imaging Archive [41,42]. The dataset was developed through a collaborative initiative involving Children’s Wisconsin, Marquette University, Varian Medical Systems, the Medical College of Wisconsin, and Stanford University. In accordance with research ethics requirements, all patient-identifiable information was removed before image processing and analysis. The usage of dataset was approved by the Institutional Review Boards of Gachon University (approval No. 1044396-202608-HR-259-01). Patient-specific volume CT dose index (CTDI_vol_) and dose-length product (DLP) values were not available in the DICOM metadata provided with the selected image series.

Eight low-dose pediatric abdominal CT datasets were randomly selected from the available cases for an exploratory methodological comparison. Detailed demographic and acquisition characteristics of the source cohort have been reported in the original dataset publication [43]. Random selection was employed to reduce intentional case-selection bias and to include variations in abdominal anatomy, contrast distribution, and image noise. The sample size was considered appropriate for an exploratory methodological evaluation involving image-specific parameter optimization, denoising, multiplanar reconstruction, and paired region of interest (ROI)-based analysis. Each dataset was processed using the original, BF, ABF, and TBF conditions, allowing direct within-case comparisons while reducing the influence of interpatient variability. The limited number of cases was considered when interpreting the findings and is acknowledged as a limitation of the study.

The original CT volumes were preprocessed using third-order spline interpolation to standardize the slice-direction resolution and improve through-plane consistency. No synthetic noise was introduced during preprocessing. Following preprocessing, BF, ABF, and TBF were applied to the axial and coronal images, and denoising performance was evaluated separately for each imaging plane. The axial plane was analyzed as the native acquisition plane, and the coronal plane as the reformat most routinely used for abdominal review, allowing us to examine whether denoising performance in the native plane is preserved after multiplanar reconstruction. The sagittal plane was not included because the coronal plane provided sufficient structural information for evaluating the hepatic parenchyma and portal vein within the scope of the present analysis. The exclusion of the sagittal plane also helped maintain the tractability of the paired, per-image-optimized analysis.

**Figure 1:**
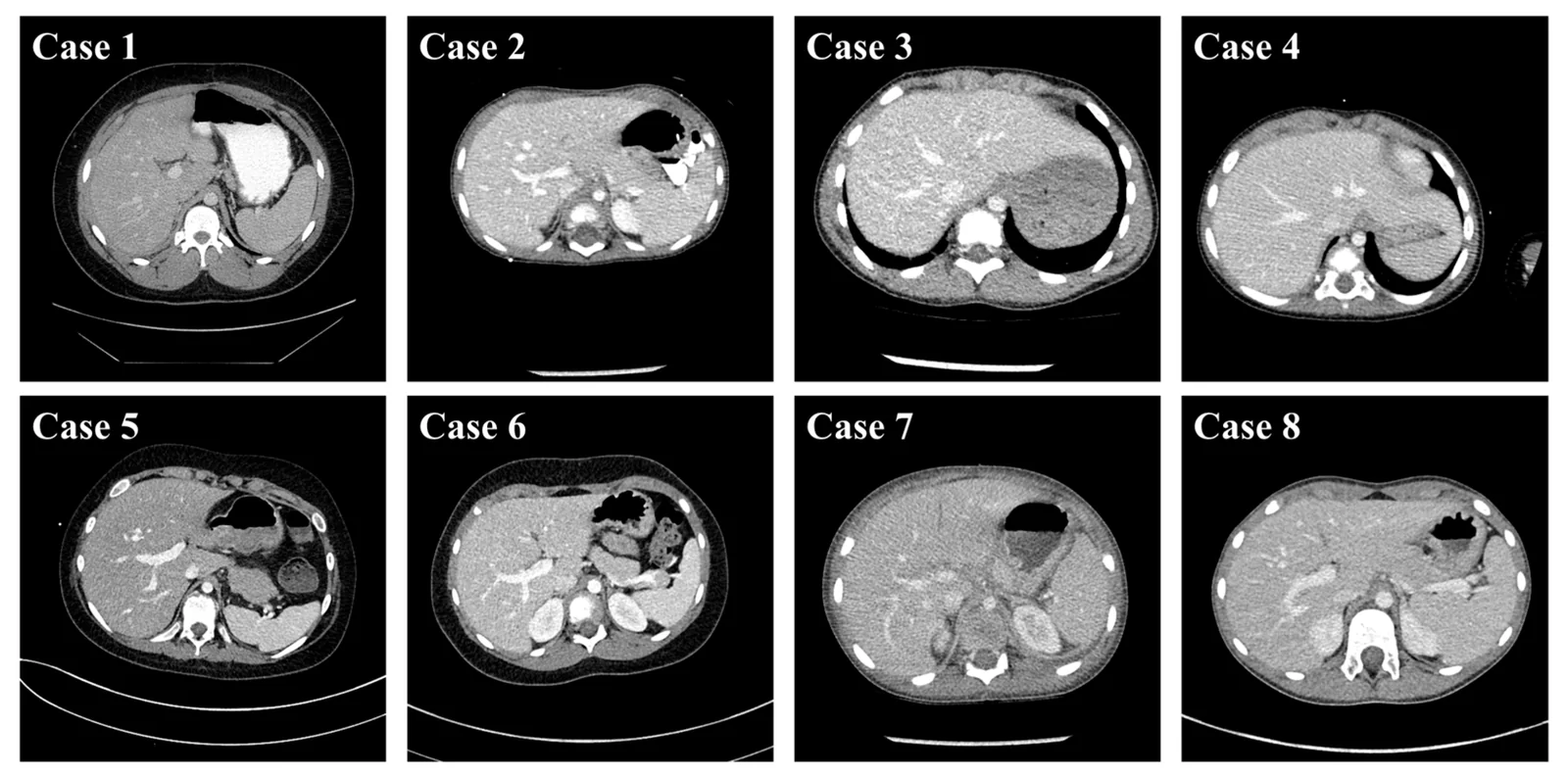
Original low-dose pediatric abdominal CT images used as inputs for the comparative evaluation of bilateral filtering-based noise reduction algorithms in the axial plane.

**Figure 2:**
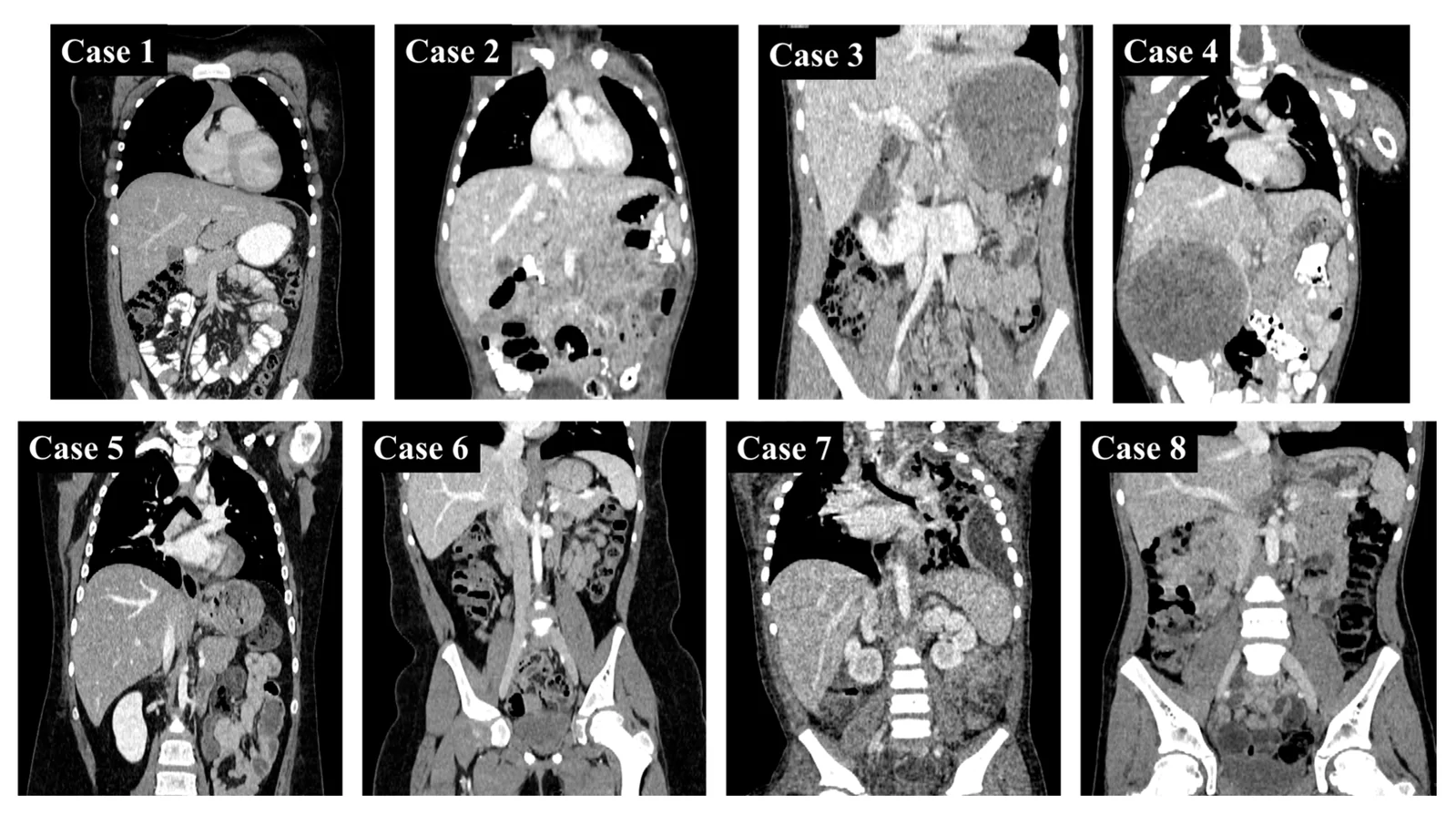
Original low-dose pediatric abdominal CT images used as inputs for the comparative evaluation of bilateral filtering-based noise reduction algorithms in the coronal plane.

The reconstructed low-dose CT images already contained visible noise associated with the original acquisition conditions and were therefore used directly as inputs for the denoising algorithms. The original low-dose pediatric abdominal CT images used for denoising are shown in Figure 1 and Figure 2.

### 2.2. Bilateral-Based Denoising Algorithms

Conventional BF is an edge-preserving smoothing technique that performs image denoising by jointly considering the spatial distance between pixels and their intensity differences [22]. The bilateral filtering processing is achieved by combining a spatial and a range domain kernel that emphasizes nearby pixels and similar intensity values, respectively. Conventional smoothing filters generally reduce noise by calculating local mean or median values within a kernel, causing substantial blurring across object boundaries. In contrast, BF theoretically suppresses image noise while preserving edge information. As a result, smoothing is preferentially performed within homogeneous regions while being inhibited across intensity discontinuities. BF was applied independently to each two-dimensional image using a window size of 5 × 5. The spatial and range kernel parameters were fixed at 1.00 and 30 Hounsfield units, respectively. The output image of the BF is computed as follows:

(1) $$I_{\mathrm{BF}}\left(p\right)=\frac{1}{W_{p}}\sum_{q\in \Omega }I\left(q\right)\exp\left(-\frac{{\left\|p-q\right\|}^{2}}{2\sigma_{s}^{2}}\right)\exp\left(-\frac{{\left|I\left(p\right)-I\left(q\right)\right|}^{2}}{2\sigma_{r}^{2}}\right)$$

where *I*(*p*) and *I*(*q*) represent the intensity values of the center pixel and a neighboring pixel, respectively. Ω denotes the local neighborhood over which the filter is applied. The parameters *σ_s_* and *σ_r_* control the spatial smoothing and sensitivity to intensity differences, respectively. The normalization factor *W_p_* is defined as the sum of all bilateral weights.

ABF is a noise reduction technique that extends conventional BF by adaptively adjusting the range parameter according to local image characteristics. In the conventional BF, a single set of spatial (*σ_s_*) and range parameters (*σ_r_*) is applied uniformly across the entire image. Although global parameterization effectively suppresses the noise in homogeneous regions, it may lead to excessive smoothing and edge blurring in areas with strong intensity variations or structural boundaries. The ABF generates a spatially varying range parameter map based on the local gradient magnitude of each pixel and incorporates this adaptive parameter into the bilateral filtering process. Consequently, the filtering strength is locally adjusted according to the underlying image content, enabling more effective noise suppression while preserving edge structures.

The ABF employed in this study extends conventional BF by adaptively adjusting the range parameter on a pixel-wise basis according to the local gradient magnitude of the input image. Previous studies have proposed adaptive bilateral filtering approaches that modify the spatial or range parameters based on local image features to overcome the performance limitations associated with the fixed parameterization of conventional BF. Building upon this concept, the ABF modeled in this study generates a spatially varying range parameter map, allowing the filtering strength to vary adaptively across homogeneous and heterogeneous regions as well as edge and transition areas.

First, the horizontal and vertical image gradients were computed to estimate the local structure variation within the input image. The gradient magnitude map was then obtained by combining two gradient components.

(2) $$G\left(x,\;y\right)=\sqrt{G_{x}{\left(x,\;y\right)}^{2}+G_{y}{\left(x,\;y\right)}^{2}}$$

where *G_x_*(*x*, *y*) and *G_y_*(*x*, *y*) denote the horizontal and vertical image gradients, respectively. Subsequently, the local range parameter, *σ_R_*(*x*, *y*), was determined according to the gradient magnitude at each pixel as follows:

(3) $$\sigma_{R}\left(p\right)=\sigma_{R,\min}+\left(\sigma_{R,\max}-\sigma_{R,\min}\right)\frac{G(x,\;y)}{G\left(x,y\right)+k_{\mathrm{grad}}+\varepsilon }$$

where *σ_R_*_,*min*_ and *σ_R,max_* denote the minimum and maximum range parameters, respectively. *k_grad_* controls the sensitivity of adaptive mapping to the gradient magnitude. *ε* means a small positive constant introduced to prevent numerical instability. Accordingly, the final formulation of the proposed ABF is given as follows:

(4) $$I_{\mathrm{ABF}}\left(p\right)=\;\frac{1}{W_{p}}\sum_{q\in \Omega }I\left(q\right)\exp\left(-\frac{{\left\|p-q\right\|}^{2}}{2\sigma_{s}^{2}}\right)\exp\left(-\frac{{\left|I\left(p\right)-I\left(q\right)\right|}^{2}}{2\sigma_{R}{\left(p\right)}^{2}}\right)$$

ABF was applied independently to each two-dimensional slice using a window size of 5 × 5. The standard deviation of the spatial kernel was fixed at 1.00. The range parameter was adaptively varied between 30 and 45 Hounsfield units according to the local gradient magnitude. A gradient sensitivity parameter of 20 was used to control spatial variation in the local range parameter. The resulting range parameter map determined the filtering strength at each pixel according to the local image structure.

The TBF is a single-image optimization-based noise reduction technique in which the filtering weights of a conventional BF are formulated as trainable parameters [36]. TBF iteratively updates the parameters by computing the residual between the input and the filtered output images. Then, the process of minimizing a loss function is defined based on the resulting residual work to reduce noise in the input image. In contrast, BF and ABF perform denoising using either predefined or empirically adjusted parameters derived from the image characteristics. This optimization enables the filtering strength to adapt to the noise level and local structural characteristics of the image. Specifically, TBF is designed to reduce intensity fluctuations within homogeneous hepatic parenchyma while minimizing structural degradation along anatomically relevant boundaries, such as portal venous vessels. For an input image *I*, TBF produces a denoising image $\hat{I}$. The difference between the input and output images is defined as the residual image *R*.

(5) $$w_{\theta }\left(p,q\right)=\exp\left(-\frac{{\left(p_{x}-q_{x}\right)}^{2}}{2\sigma_{x}^{2}}-\frac{{\left(p_{y}-q_{y}\right)}^{2}}{2\sigma_{y}^{2}}\right)\exp\left(-\frac{{\left(I\left(p\right)-I\left(q\right)\right)}^{2}}{2\sigma_{r}^{2}}\right)$$

Here, *p* and *q* denote the coordinates of the center pixel and a neighboring pixel within the local window Ω, respectively. *I*(*p*) and *I*(*q*) represent the corresponding intensity values. The first exponential term corresponds to the spatial-domain kernel, where *σ_x_* and *σ_y_* indicate the standard deviations of the spatial Gaussian kernel along the *x* and *y* directions, respectively. The second exponential term corresponds to the range-domain kernel, in which *σ_r_* denotes the standard deviation controlling the sensitivity to intensity differences. Unlike the conventional BF, in which these parameters are fixed or empirically determined, *σ_x_*, *σ_y_*, and *σ_r_* are treated as trainable parameters *θ* in the TBF, allowing the filter to be optimized in a data-driven manner. The spatial kernel parameters *σ_x_* and *σ_y_* were initialized to 1.00, and the range kernel parameter *σ_r_* was initialized to 0.01 after scaling the input images to the range from 0 to 1.

(6) $$R=I-\hat{I}$$

(7) $$I_{\mathrm{TBF}}\left(p\right)=\frac{1}{W_{p}}\sum_{q\in \Omega }I\left(q\right)w_{\theta }\left(p,\;q\right)$$

(8) $$L\left(\theta \right)=\frac{1}{N}\sum_{p}{\left(I_{\mathrm{input}}\left(p\right)-I_{\mathrm{TBF}}\left(p\right)\right)}^{2}$$

where $L\left(\theta \right)$ denotes the mean squared error loss function computed between the input image *I_input_*(*p*) and the filtered output *I_TBF_*(*p*) over all *N* pixels, and is used to guide the optimization of the trainable parameters *θ*. The parameters *θ* = {*σ_x_*, *σ_y_*, *σ_r_*} are iteratively updated using the Adam optimizer, where the learning rate *η* was set to 0.1 and the exponential decay rates for the first and second moment estimates were set to 0.9 and 0.999, respectively. The gradient of the loss with respect to trainable parameters was computed via backpropagation. Optimization was performed for a fixed number of 1000 iterations set as default.

(9) $$\theta \leftarrow \theta -\eta \nabla_{\theta }L\left(\theta \right)$$

### 2.3. Qualitative and Quantitative Evaluation

Both qualitative and quantitative evaluations were performed to assess denoising performance. For the qualitative evaluation, the original BF-, ABF-, and TFB-processed images were assessed independently by three experts, each with more than five years of experience in medical image processing, using predefined criteria including noise homogeneity within the liver parenchyma, preservation of edges and vessel boundaries, and the presence of blurring of spurious structures.

The original low-dose pediatric abdominal CT images were compared with the corresponding BF-, ABF-, and TBF-applied images to quantitatively evaluate noise reduction and image quality. ROI-based measurements were performed independently in the axial and coronal planes. The liver parenchyma and portal vein were selected as complementary and well-defined targets for evaluating noise reduction and contrast preservation. The liver parenchyma provides a large and relatively homogeneous soft tissue region suitable for assessing local signal variability using CV. In contrast, the portal vein represents an adjacent vascular structure with a signal level distinct from the surrounding liver tissue.

Preservation of contrast between the portal vein and liver parenchyma is clinically relevant in terms of identifying intrahepatic vascular structures and assessing their anatomical boundaries. Therefore, CNR evaluation was used to determine whether noise reduction achieved without compromising soft tissue contrast. The combine evaluation of both regions allowed characterization of the trade-off between noise suppression and structural contrast preservation.

**Figure 3:**
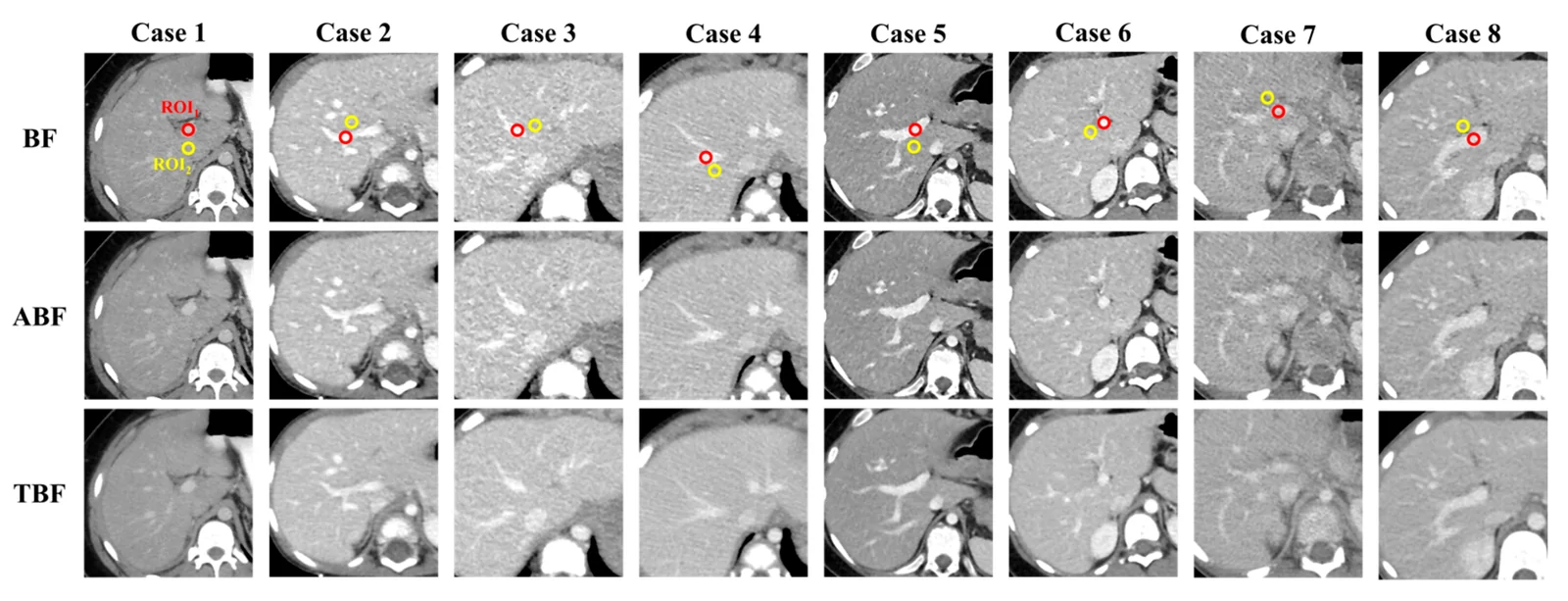
The magnified results for visual comparison of axial pediatric low-dose CT images after application of the bilateral filter (BF), adaptive BF (ABF), and trainable BF (TBF). Columns represent Case 1–8 from left to right, whereas rows indicate the results obtained using BF, ABF, and TBF from top to bottom. Region of interests were marked with red and yellow circles for portal vein and liver parenchyma, respectively.

**Figure 4:**
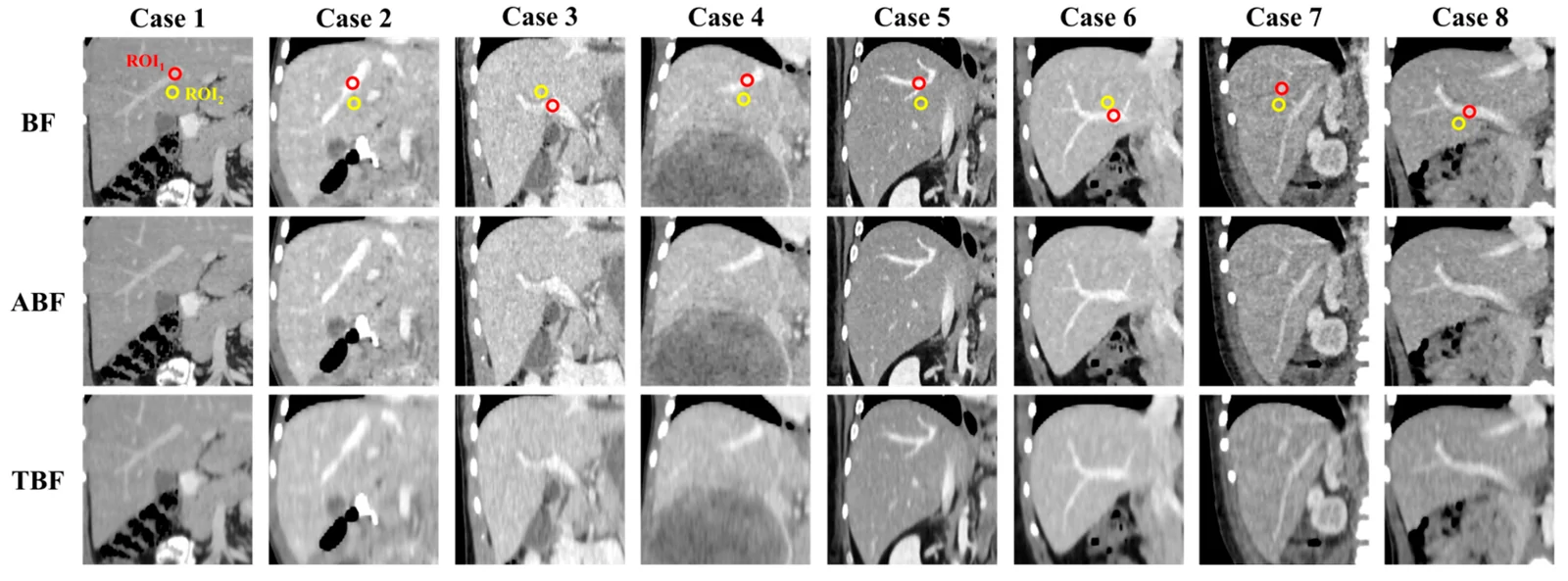
The magnified results for visual comparison of coronal pediatric low-dose CT images after application of the bilateral filter (BF), adaptive BF (ABF), and trainable BF (TBF). Columns represent Case 1–8 from left to right, whereas rows indicate the results obtained using BF, ABF, and TBF from top to bottom. Region of interests were marked with red and yellow circles for portal vein and liver parenchyma, respectively.

For each case, one circular ROI with a fixed diameter of 1 cm was placed within homogeneous liver parenchyma while avoiding vessels and artifacts. A second circular ROI of the same size was positioned within the portal vein. Each ROI was restricted to a single tissue type, as indicated by the red and yellow regions in Figure 3 and Figure 4. The same ROI diameter was applied to all cases. Measurements were obtained from one representative slice in each imaging plane, resulting in one liver ROI and one portal vein ROI for each case and plane. ROI location and size were kept identical across the original, BF, ABF, and TBF image sets within each imaging plane. The mean pixel value and standard deviation within each ROI were measured to calculate the contrast-to-noise ratio (CNR) and coefficient of variation (CV), which were used to assess the changes in image characteristics according to the application of each algorithm [44,45].

(10) $$\mathrm{CNR}=\frac{\left|\mu_{\mathrm{ROI}1}-\mu_{\mathrm{ROI}2}\right|}{\sqrt{\sigma_{\mathrm{ROI}1}^{2}+\sigma_{\mathrm{ROI}2}^{2}}}$$

(11) $$\mathrm{CV}=\frac{\sigma_{\mathrm{ROI}2}}{\mu_{\mathrm{ROI}2}}$$

Here, *σ_ROI_*_1_ and *σ_ROI_*_2_ denote the standard deviation of ROIs set in portal vein and liver, respectively. *μ_ROI_*_1_ and *μ_ROI_*_2_ indicate the mean of ROIs set in portal vein and liver, respectively.

### 2.4. Statistical Analysis

Statistical analyses were conducted using a repeated measures design because each of the eight cases was evaluated under the original, BF, ABF, and TBF conditions. The four conditions were compared separately for each imaging plane and quantitative measurements. The CV and CNR were designated as the primary image quality outcomes, whereas the mean value and standard deviation measured within the ROI were analyzed as secondary component measures. The Friedman test was selected because of the limited sample size, which precluded a reliable assessment of the normality of within–case differences. Pairwise comparisons between the four conditions were performed using two-sided Wilcoxon signed-rank tests when the Friedman test indicated a significant overall difference. Holm-adjustment was applied separately to the six pairwise comparisons within each combination of imaging plane and quantitative measures. Kendall’s coefficient of concordance, W, was reported as an effect size and an indicator of the consistency of method rankings across cases. Statistical significance was defined as a Holm-adjusted *p*-value ≤ 0.05. All analyses were performed using Python version 3 (Python software foundation, Beaverton, OR, USA) with SciPy version 1.17.0.

## 3. Results

### 3.1. Qualitative Evaluation of Bilateral-Based Noise Reduction Algorithms

Visual comparison of the axial and coronal images showed that BF, ABF, and TBF reduced the noise observed in the original condition. The results obtained after application of each algorithm are presented in Figure 3 and Figure 4, which correspond to the axial and coronal planes, respectively. The resulting axial and coronal images showed consistent differences in noise reduction and structural preservation across the three bilateral-based noise reduction methods.

BF preserved fine hepatic texture, vascular boundaries, and local anatomical details, although noticeable noise remained within homogeneous liver regions. ABF further improved parenchymal uniformity while maintaining the visibility of major vessels and tissue boundaries, providing a balanced appearance between noise reduction and detail preservation. TBF produced the most pronounced visual noise reduction and the most homogeneous liver parenchyma in both imaging planes. However, over-smoothing reduced fine tissue texture and softened the boundaries of small vascular structures. In selected cases, local signal patterns after TBF processing appeared indistinct from anatomical structures. Moreover, discontinuous segments of the portal vein appeared to be connected after TBF processing, which may represent a filtering-induced artifact in Case 5 of Figure 3.

The differences were more evident in the axial plane, whereas through-plane interpolation and partial-volume effects in the coronal plane further reduced the sharpness. The overall qualitative trends were similar across both orientations despite these plane-specific characteristics.

### 3.2. Quantitative Evaluation

**Figure 5:**
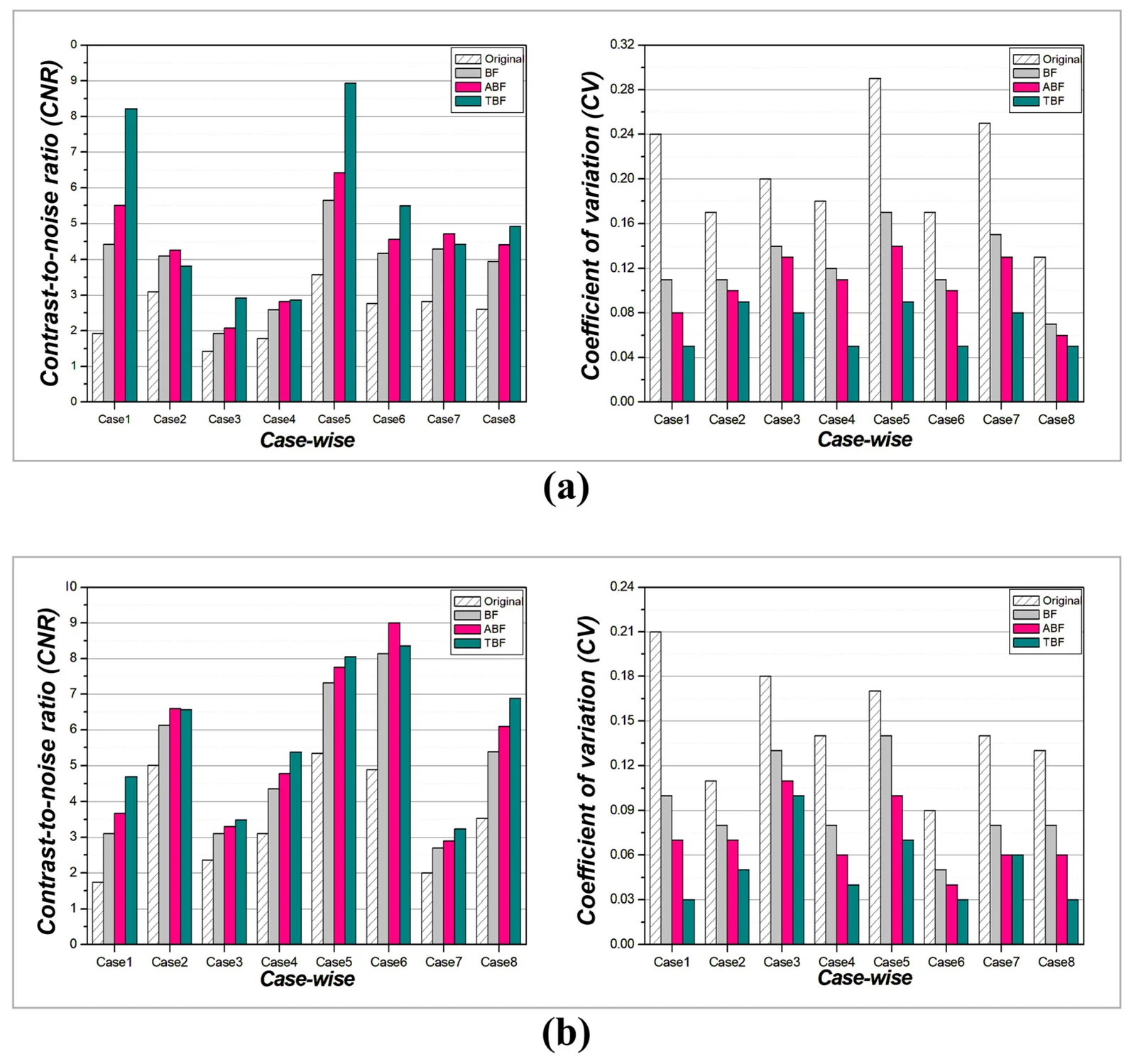
Case-wise quantitative comparison using contrast-to-noise ratio and coefficient of variation in the original, bilateral filter (BF), adaptive BF (ABF), and trainable BF (TBF) conditions in (**a**) the axial and (**b**) coronal planes.

CNR and CV were evaluated in the axial and coronal planes across eight cases. CNR represented the distinction between the liver parenchyma and portal vein, whereas CV served as a surrogate measure of noise within homogeneous liver tissue. Figure 5 presents the case-specific values under the original, BF, ABF, and TBF conditions.

In the axial plane, CNR increased from 2.50 ± 0.73 in the original condition to 3.89 ± 1.15, 4.35 ± 1.38, and 5.21 ± 2.28 after BF, ABF, and TBF, respectively. The CV decreased from 0.210 ± 0.054 to 0.128 ± 0.031, 0.111 ± 0.028, and 0.071 ± 0.019. The coronal plane showed the same overall pattern, with CNR rising from 3.50 ± 1.43 to 5.16 ± 2.24, 5.52 ± 2.22, and 5.84 ± 1.96, and the CV declining from 0.152 ± 0.040 to 0.097 ± 0.032, 0.078 ± 0.023, and 0.057 ± 0.024. Overall, TBF yielded the lowest average CV and the highest average CNR across both planes, although ABF achieved the highest CNR in Cases 2 and 7. All three noise reduction methods reduced the CV and increased the CNR relative to the original condition across both imaging planes. The average measurements favored TBF, whereas the ordering of ABF and TBF varied in selected cases.

### 3.3. Statistical Comparison of Quantitative Metrics Across Denoising Methods

**Figure 6:**
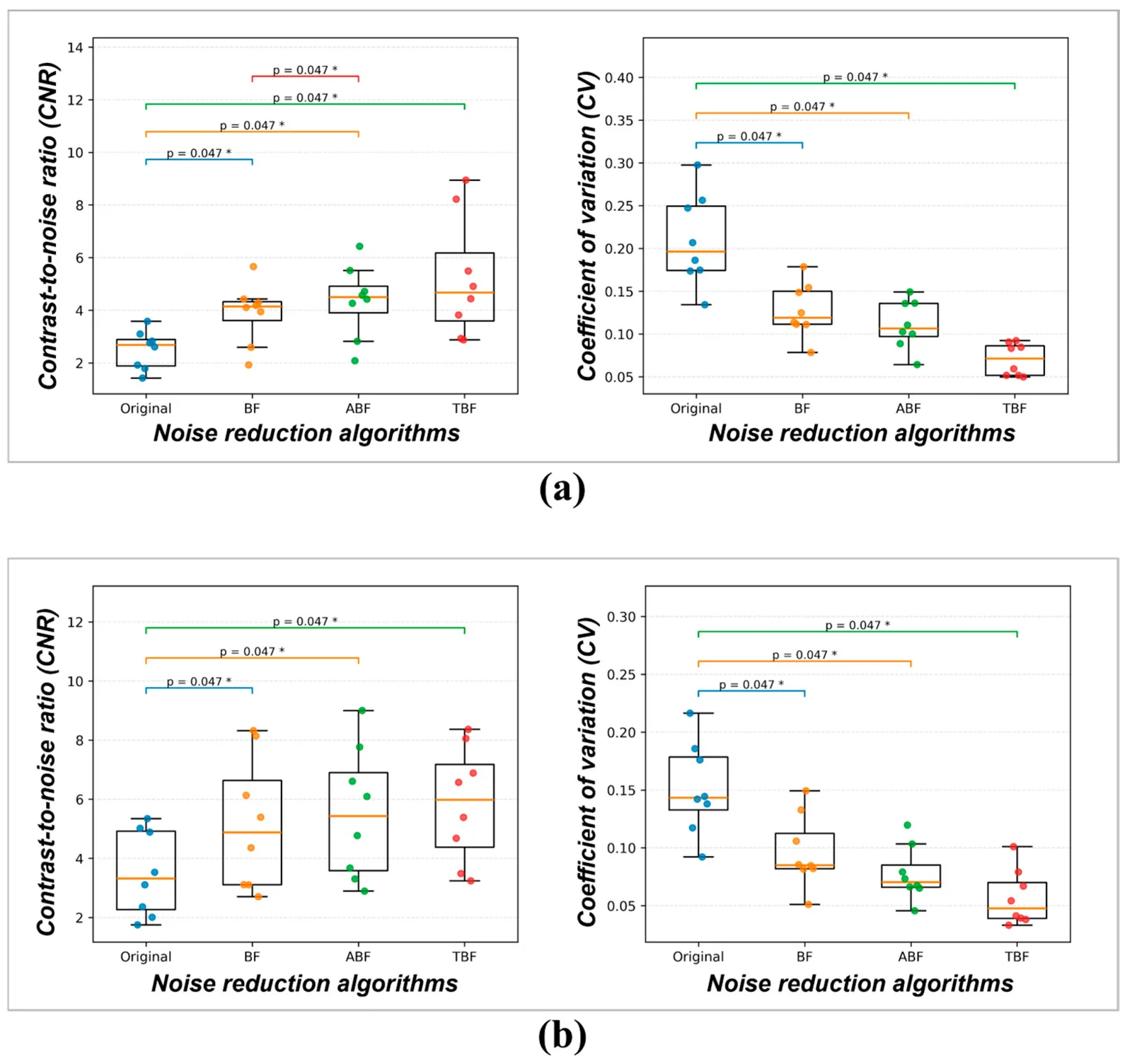
Statistical comparison of contrast-to-noise ratio (CNR) and coefficient of variation (CV) across the original, bilateral filter (BF), adaptive BF (ABF), and trainable BF (TBF). Results are presented for (**a**) the axial plane and (**b**) the coronal plane with CNR shown on the left and CV on the right. The asterisk (*) indicates the statistical significance (*p* < 0.05). Each circle represents the distribution of quantitative evaluation results of the original, BF, ABF, and TBF with the color of blue, orange, green, and red, respectively.

Figure 6 presents the distributions of CNR and CV across the four conditions in the axial and coronal planes, together with selected Holm-adjusted pairwise comparisons. Box plots represent the median and interquartile range, and the overlaid points indicate individual measurements from the eight cases. Brackets indicate statistically significant pairwise comparisons based on two-sided Wilcoxon signed-rank tests with Holm-adjustment after a significant Friedman test.

The Friedman test demonstrated significant differences in CV among the four conditions in both imaging planes. Complete agreement in method rankings was observed across the eight cases, with Kendall’s W equal to 1.00. All six pairwise comparisons remained significant after Holm adjustment. The standard deviations measured within the liver and portal vein ROIs showed a similar pattern, indicating a consistent reduction in local signal variability after denoising.

The CNR also differed significantly among the four conditions in the axial and coronal planes. Each noise reduction method produced a significantly higher CNR than the original condition. However, pairwise differences among BF, ABF, and TBF were less consistent. In the axial plane, a significant difference was observed between BF and ABF, whereas neither comparison involving TBF reached statistical significance. In the coronal plane, none of the pairwise comparisons among the three denoising methods remained significant after Holm adjustment. These results were reflected in the wider overlap of the CNR distributions among the filtered conditions, particularly in the coronal plane.

The mean signal value within the liver ROI remained statistically comparable across the four conditions in both imaging planes as shown in Table 1. In contrast, the portal vein ROI showed significant changes that were limited to comparisons involving TBF. In the coronal plane, all six pairwise comparisons remained significant after Holm-adjustment. These findings indicate more consistent changes in the mean signal value of the portal vein ROI than in the liver ROI.

## 4. Discussion

The central challenge in pediatric CT is that radiation exposure carries a disproportionately greater long-term risk in children than in adults, while dose reduction directly degrades the image quality required for diagnostic assessment [46]. Dose reduction is clinically mandated under the ALARA principle because pediatric tissues exhibit greater radiosensitivity [47]. This requirement limits the use of increased radiation exposure as a means of compensating for noise that occurs during the CT image acquisition process [48]. Consequently, greater emphasis must be placed on post-processing methods that improve the quality of low-dose images.

Lowering the tube current or voltage reduces photon flux and increases mixed Gaussian–Poisson noise in reconstructed images [49]. The influence of noise is compounded in pediatric patients by their smaller body habitus and inherently lower soft-tissue contrast. Both characteristics narrow the margin for reliable detection of subtle pathological findings. Denoising performance should therefore be evaluated according to the specific requirements of pediatric imaging. IR operates directly on raw projection data and remains dependent on vendor-specific reconstruction pipelines, limiting its application to previously reconstructed clinical images. Deep learning-based denoising networks have demonstrated strong quantitative performance. However, large and carefully curated training datasets remain scarce in pediatric radiology because of smaller patient populations and stricter data-sharing constraints [50,51,52]. Opaque model behavior also raises concerns regarding the generation of anatomically nonexistent structures, particularly when subtle findings carry substantial diagnostic importance [53,54].

The bilateral-based approaches evaluated in this study represent a progression from explicit parameter control to image-specific optimization. BF uses fixed spatial and range parameters, whereas ABF adjusts the range parameter according to a predefined gradient-based rule. TBF retains the bilateral weighting framework while directly optimizing both parameters through gradient-based learning. Therefore, BF and ABF provide relatively interpretable filtering behavior, whereas TBF offers greater adaptability and requires less dependence on external training datasets than conventional deep learning networks. Consequently, TBF occupies an intermediate position between classical bilateral filtering and learning-based denoising.

All three denoising methods reduced image noise compared to the original condition. CV generally decreased from BF to ABF and TBF, whereas CNR showed an overall increase in the same sequence. Pooled statistical analysis confirmed significant and consistent reductions in CV and ROI standard deviations across both imaging planes. Each denoising method also produced significantly higher CNR than the original condition. However, incremental CNR differences among BF, ABF, and TBF were less consistent. A significant difference between BF and ABF was observed only in the axial plane, while comparisons involving TBF remained nonsignificant. In the coronal plane, no pairwise comparison among the three denoising methods retained statistical significance after Holm-adjustment.

The effect size estimates provided additional information beyond statistical significance. Kendall’s W reached 1.00 for CV in both imaging planes, indicating identical ordering of the four conditions across all eight cases. The corresponding values for the liver and portal vein ROI standard deviations ranged from 0.87 to 1.00, demonstrating that the reduction in local signal variability occurred consistently across the dataset, rather than being driven by a small subset of cases. In comparison, Kendall’s W values for CNR were 0.84 in the axial plane and 0.79 in the coronal plane. These values indicate strong overall concordance, although lower consistency reflects case-specific changes in method ranking.

The significant Friedman results for CNR indicate that at least one of the four conditions differed, rather than establishing a uniform performance hierarchy among BF, ABF, and TBF. Significant comparisons between each denoising method and the original condition demonstrated a clear overall benefit of post-processing. In contrast, the nonsignificant comparisons among the filtered conditions indicate that the additional CNR gain associated with greater filtering complexity was smaller and more dependent on individual image characteristics. Accordingly, the statistical findings support the effectiveness of denoising in general more strongly than the superiority of a single filtering method.

Component-level analysis helps explain this distinction. The mean pixel value within the portal vein ROI differed significantly among the four conditions at the group level, whereas the corresponding liver measurements remained statistically stable. The consistent reduction in ROI standard deviations demonstrates reliable suppression of local signal variability. However, changes in the mean pixel value influenced the numerator of the CNR calculation and occasionally offset the benefit of reduced noise. Consequently, a statistical improvement in CV or ROI standard deviation provides no guarantee of proportional gains in tissue contrast or quantitative signal preservation.

Cases 2 and 7 illustrate the interaction between noise reduction and signal preservation. TBF reduced the standard deviation within the liver parenchymal ROI, whereas the accompanying decrease in the mean signal value narrowed the contrast difference between the liver and portal vein. ABF therefore achieved a higher CNR in these cases by preserving the inter-ROI signal difference more effectively, despite providing less pronounced noise suppression than TBF. These findings indicate that a lower CV alone is insufficient to establish better tissue contrast. Evaluation of denoising performance should therefore consider local signal variability together with the stability of mean ROI values.

The signal changes observed with TBF appear to be related to its image-specific optimization process. The optimized spatial and range parameters determine the effective filtering neighborhood and the relative contribution of surrounding pixels. In selected images, increased weighting of neighboring pixels with lower signal values appeared to reduce the mean pixel value within the liver ROI. An optimization objective focused primarily on noise reduction could favor parameter combinations that suppress local variation without explicitly preserving tissue-specific CT numbers. This behavior provides a plausible explanation for the stronger smoothing, local signal shifts, and occasional reduction in CNR observed with TBF.

The structural distortion associated with these signal changes, including the over-smoothing and blurring observed on visual assessment, was not objectively quantified in the present study. Full-reference indices such as structural similarity index measure and feature similarity index measure are most reliably computed against a noise-free reference image which is unavailable in clinical dataset. However, reference-free measures such as the edge preservation index, which can be computed between the denoised and input images, could provide an objective characterization of TBF-induced structural distortion. The incorporation of above-mentioned quantitative metrics in future studies would provide a more comprehensive assessment of structural preservation and complement the qualitative and ROI-based findings of this study.

Image-specific optimization also introduces a practical trade-off. The spatial and range parameters are optimized separately for each input image, and processing time varies according to image content, initialization, learning rate, and stopping criteria. Different inputs also produce different final parameter values. Furthermore, the computational burden of TBF was substantial. Experiments were performed on a workstation equipped with an AMD Ryzen 7 9800X3D 8-core CPU (Advanced Micro Devices, Inc., Santa Clara, CA, USA), an NVIDIA GeForce RTX 5080 GPU (NVIDIA corporation, Santa Clara, CA, USA), and 32 GB of RAM (Samsung Electronics Co., Ltd., Suwon, Republic of Korea). The non-iterative BF and ABR required approximately 37 and 16 s per volume, respectively. In contrast, TBF consumed about 850 s per volume on average because gradient-based optimization was performed independently for each slice. At the volume level, TBF was approximately 20- to 50- fold slower than BF and ABF. The increased computational demand originates from the per-image optimization procedure TBF and may limit practical implementation in time-sensitive clinical workflows. Although this flexibility supports adaptation to individual noise patterns, insufficient standardization of the optimization procedure limits reproducibility across images and institutions. Further development of TBF should therefore incorporate constraints related to CT number stability, structural preservation, and standardized convergence criteria.

The differences between the axial and coronal imaging planes warrant further consideration. Coronal images are more susceptible to through-plane interpolation, partial-volume effects, and changes in the apparent orientation of vascular structures. These factors influence local signal variability and sharpness of the portal vein boundary. Differences in CNR improvement between the two planes likely reflect both the performance of each denoising method and the image characteristics associated with multiplanar reconstruction. Evaluation across multiple planes is particularly important because favorable performance in the native axial plane provides no guarantee of equivalent contrast and structural preservation in reformatted images.

Although the axial and coronal analyses produced different pairwise significance patterns, separate within-plane tests provide insufficient evidence to establish a significant difference in filtering effects between the two planes. Confirmation of such a difference requires a formal method-by-plane interaction analysis. Accordingly, the present findings represent plane-specific response patterns rather than a statistically confirmed difference in algorithm performance between image orientations.

The qualitative findings complemented the quantitative results. BF preserved contrast-enhanced gastric regions, vascular boundaries, and fine anatomical structures more clearly than TBF, although substantial residual noise remained within homogeneous tissues. ABF produced a more uniform liver parenchyma with less visible graininess despite a similar overall level of noise reduction as BF. These observations suggest that ABF provides a more balanced compromise between homogeneous region smoothing and structural detail preservation. In contrast, TBF produced the most pronounced noise suppression and the most favorable average CV and CNR values. However, TBF induced severe blurring, reduced boundary definition, and occasional structures that were difficult to distinguish from processing artifacts.

The present findings should be interpreted within the scope of an exploratory and methodological comparison. Even within this scope, the study yielded a clear and internally consistent results under a fully paired within-case design in which each case was processed under all four conditions at identical ROI positions. Consequently, every case served as its own control, thereby eliminating inter-patient variability from the primary comparisons. This design produced highly concordant rankings across the eight cases, reflected in Kendall’s W values of 1.00 for CV in both planes and 0.79 to 1.00 for the ROI-level measures, indicating that the observed ordering was reproduced across all cases rather than being driven by outliers. The consistency of these results supports the robustness of the principal performance tendencies identified for BF, ABF, and TBF. Building on these consistent findings, future studies with larger pediatric cohorts are warranted to confirm the robustness of the observed effects and improve the generalizability of these methods for clinical application.

Consistent with this limitation, several significant pairwise comparisons yielded the same Holm-adjusted *p*-value of 0.0469. This pattern reflects the discrete resolution of the exact Wilcoxon signed-rank test with eight paired observations rather than identical magnitudes of difference. Although these comparisons satisfied the predefined significance threshold, the adjusted values remained close to 0.05. Therefore, interpretation should emphasize the consistent direction of change and the accompanying Kendall’s W estimates rather than the *p*-values alone.

Inferential emphasis should remain on CV and CNR as primary image quality outcomes because several ROI-derived measures were examined. The mean pixel values and standard deviations were interpreted more appropriately as component measures that explained changes in the primary metrics. Quantitative evaluation was also limited to manually placed ROIs and provided limited information regarding noise texture, spatial resolution, edge preservation, and absolute CT number stability.

An additional limitation concerns the availability of radiation dose information in the source dataset. Patient-specific CTDI_vol_ and DLP values were not available in the DICOM metadata of the image series included in the analysis. Therefore, direct assessment of the relationship between radiation dose and denoising performance was not possible. Future studies using datasets with complete dose metadata should evaluate denoising performance across different radiation dose levels.

The findings should be regarded as preliminary because the study included only eight cases and did not include lesion-level evaluation or a formal radiologist reader assessment. Definitive clinical recommendations will require validation in larger pediatric cohorts with lesion-level evaluation and complementary image quality measures. Blinded radiologist reader assessments will also be necessary to establish the clinical relevance of the observed quantitative improvements. Further algorithm refinement will focus on maintaining effective noise suppression while preserving quantitative CT values and anatomical fidelity.

## 5. Conclusions

In this study, three bilateral-based noise reduction strategies, BF, ABF, and TBF, were applied to pediatric low-dose abdominal CT images and evaluated in the axial and coronal planes. Image quality was assessed through visual comparison and quantitative analysis using CV and CNR. All three methods reduced image noise compared with the original images. BF relatively preserved fine anatomical structures, retaining residual noise, whereas ABF achieved a more balanced performance between noise reduction and structural preservation. TBF provided the strongest overall noise suppression and the most favorable quantitative results, although greater blurring and a hallucinated structure were observed. These findings demonstrate the potential of bilateral-based noise reduction strategies to improve the quality of pediatric low-dose CT images. The results should be regarded as preliminary and require validation in larger studies before clinical adoption as they were obtained from a limited exploratory cohort. Further refinement is required to ensure clinical reliability without compromising quantitative accuracy or anatomical details.

## Figures and Tables

**Table 1:** Friedman test and post hoc Wilcoxon signed-rank comparisons among the original, BF, ABF, and TBF conditions. Adjusted *p*-values were obtained using the Holm method across the six pairwise comparisons within each combination of imaging plane and quantitative evaluation metric.

Plane	Metric	χ^2^ (3)	*p*-Value	Kendall’s W
Axial	PV SD	24.00	<0.001	1.00
	PV mean	19.05	<0.001	0.79
	Liver SD	24.00	<0.001	1.00
	Liver mean	3.75	0.290	0.16
	CNR	20.25	<0.001	0.84
	CV	24.00	<0.001	1.00
Coronal	PV SD	20.85	<0.001	0.87
	PV mean	24.00	<0.001	1.00
	Liver SD	24.00	<0.001	1.00
	Liver mean	3.00	0.392	0.13
	CNR	19.05	<0.001	0.79
	CV	24.00	<0.001	1.00

PV, portal vein; SD, standard deviation; CNR, contrast-to-noise ratio; CV, coefficient of variation.

## Data Availability

The data used in this study were obtained from The Cancer Imaging Archive (TCIA), a publicly accessible repository of medical images (https://www.cancerimagingarchive.net/ (accessed on 5 March 2026)). The specific dataset (Pediatric-CT-SEG) is available for unrestricted research use in accordance with TCIA data usage policies. The datasets generated during and/or analyzed during the current study are available from the corresponding author on reasonable request.

## Abbreviations

CT	Computed tomography
ALARA	As Low As Reasonably Achievable
BF	Bilateral filter
ABF	Adaptive bilateral filter
TBF	Trainable bilateral filter
ROI	Region of interest
CNR	Contrast-to-noise ratio
CV	Coefficient of variation
